# Changes in VEGF-related factors are associated with presence of inflammatory factors in carbohydrate metabolism disorders during pregnancy

**DOI:** 10.1371/journal.pone.0220650

**Published:** 2019-08-15

**Authors:** Masahiko Sugimoto, Mineo Kondo, Yuki Kamimoto, Tomoaki Ikeda, Alecia Cutler, Ali Mariya, Bela Anand-Apte

**Affiliations:** 1 Department of Ophthalmology, Mie University Graduate School of Medicine, Tsu, Japan; 2 Department of Obstetrics and Gynecology, Mie University Graduate School of Medicine, Tsu, Japan; 3 Department of Ophthalmic Research, Cole Eye Institute, Cleveland Clinic Foundation, Cleveland, Ohio, United States of America; University of Mississippi Medical Center, UNITED STATES

## Abstract

The aim of this study was to determine the action of molecules in carbohydrate metabolism disorders during pregnancy. The concentration of different types of cytokines and vascular endothelial growth factor (VEGF) in the plasma were measured in 4 groups of women: Group I, normal pregnancy (n = 10); Group II, patients with gestational DM (n = 12); Group III, pregnant patients with preexisting DM (n = 16); and Group IV, diabetic non-pregnant women (n = 22). The plasma VEGF concentration was significantly higher in the women in Group IV than in other groups (*P* <0.01). The concentration of the soluble form of the VEGF receptor-1 (sVEGFR-1) was significantly higher in Group I than in other groups (*P* <0.01). The concentration of soluble form of the VEGF receptor-2 (sVEGFR-2) was significantly lower in Groups I than in other groups (*P* <0.05). The concentrations of monocyte chemotactic protein-1 (MCP-1) and eotaxin were significantly lower in Group I than in Groups III and IV. The levels of interleukin (IL)-8, IL-6, and tumor necrosis factor-α (TNF-α) were significantly higher in Group I than in Group IV. Both the VEGF-related molecules and the Inflammatory cytokines are altered in pregnant women with the carbohydrate metabolism disorders.

## Introduction

The systemic disorders that develop during pregnancy are strongly associated with changes of the micro circulation. Patients with diabetes mellitus (DM) are known to develop complications during pregnancy [1], and this clinical state is called “carbohydrate metabolism disorders during pregnancy (CMDP)”. The complications occur both in patients with preexisting diabetes mellitus (pDM) and with “hyperglycemic disorder in pregnancy” including gestational diabetes mellitus (GDM). GDM is defined as any degree of glucose intolerance after the first recognition of pregnancy [2] in contrast to pDM. The diabetes-associated complications include congenital abnormalities of the newborn, fetal macrosomia, and systemic disorders that are detected during the neonatal stage. Strict blood sugar control is recommended to prevent these complications [3].

Micro-circulatory changes during pregnancy also leads to a number of broad-ranging alterations of the vasculature. Vascular endothelial growth factor (VEGF) plays an important role in vascular angiogenesis, inducing tumor growth, inflammatory diseases, and ischemic diseases. [4–6]. The development of preeclampsia and the progression of diabetic retinopathy (DR) are known complications during CMDP and VEGF is also known to play important roles in these processes [7–12]. VEGF interact with two transmembrane receptors, VEGFR-1 and VEGFR-2, and the interactions trigger an intracellular tyrosine kinase cascade. Then these VEGF-related molecules stimulate the vascular endothelial cells resulting in an increase in the vascular permeability [13, 14]. Both types of receptors are expressed in vascular endothelial cells, and they play important roles. The soluble form of VEGFR-1 (sVEGFR-1) is produced by alternative splicing with the same gene encoding the VEGFR-1. Circulating sVEGFR-1 binds to VEGF with high affinity and neutralizes the VEGF activity [15]. During development, sVEGFR-1 functions primarily as a non-signaling resolver that suppresses the activity of VEGF [16]. Thus, sVEGFR-1 is known as an endogenous inhibitor of angiogenesis. The soluble form of VEGFR-2 (sVEGFR-2) is also known to inhibit the VEGF-induced endothelial cell proliferation and has antiangiogenic properties similar to sVEGFR-1 [17]. However, the affinity and activity of sVEGFR-2 is stronger than that of sVEGFR-1. sVEGFR-2 influences the normal endothelial cell function, vascular permeability, and angiogenesis more than sVEGFR-1 [18,19]. These VEGF-related molecules also play important roles during pregnancy and fetal development. Mutations of VEGF is lethal for embryos because vasculogenesis is impaired resulting in abnormal development [20].

There are many reports concerning the plasma VEGF levels during normal pregnancy. In normal pregnancy, low concentrations of sVEGFR-1 allow for proper VEGF signaling, and the function of the endothelial cells is maintained. However, once complication have developed, e.g., hypertensive pregnancy and preeclampsia, the maternal plasma VEGF levels are changed [9–12]. An increased production of sVEGFR-1 by the placenta leads to a decrease in the bioavailable VEGF which then causes a dysfunction of the endothelial cells [21]. So, the levels of VEGF and sVEGFR-1 are strongly related to the maintenance of normal pregnancy.

Endothelial cell disfunction is the principal complication of diabetes. The results of earlier studies have shown the possibility of vascular endothelial cell dysfunction in CMDP [22, 23]. Inflammation also plays an important role in endothelial cell disfunction, and there are some reports on an elevation of circulating inflammation markers in women with GDM [24, 25]. From these backgrounds, the mechanisms underlying the activity of CMDP may differ from that during regular DM or pregnancy. But the relationships between the VEGF-related molecules and inflammatory related molecules on CMDP have not been definitively determined. Thus, the purpose of this study was to determine whether the background VEGF-related molecules and other inflammation-related molecules are altered in patients with CMDP.

## Patients and methods

The study was carried out with the approval of the Institutional Ethics Committee (Approval #2290) and was also registered at http://www.umin.ac.jp (UMIN ID 000033765). The procedures conformed to the tenets of the Declaration of Helsinki. A written informed consent was obtained from all participants. Women with CMDP and normal pregnancy were recruited from the Department of Obstetrics and Gynecology of the Mie University Hospital. Women with diabetes were recruited from the Department of Endocrinology, Mie University Hospital. The subjects were examined at the Department of Ophthalmology during the gestational period. The clinical histories of all patients were obtained from their medical records.

### Sample collection

Sixty women were studied, and they were classified into three groups; Group I included 10 women with normal pregnancy; Group II included 12 women with GDM; and Group III included 16 women with preexisting diabetes. Group IV was the control group and included 22 diabetic non-pregnant women. The gestational week was estimated based on the findings of early ultrasound scanning.

### Inclusion and exclusion criteria

The inclusion criteria were women who were ≥20- or <50-years-of-age. The exclusion criteria were; history of systemic disease requiring medications that could affect the results, severe renal failure with creatinine ≥2.0 mg/dl or >Stage IIb of nephropathy defined by the classification of diabetic nephropathy, poorly controlled hypertension with systolic BP >200 mmHg or diastolic BP >110 mmHg, the development of complications such as preeclampsia, pregnancy-induced hypertension, and patients who were judged ineligible for other reasons determined the investigators.

### Normal pregnancy (Group I)

Non-diabetic pregnant women who were being followed in the Department of Obstetrics and Gynecology were studied. Normal pregnancy was defined as pregnancy without any general complications, and none of these patients had ocular complications.

### Gestational diabetes (GDM; Group II)

GDM women who were being followed in the Department of Obstetrics and Gynecology were studied. GDM was diagnosed to be present when any degree of glucose intolerance was first detected with the 75 g oral glucose tolerance test during pregnancy.

### Preexisting diabetes (Group III)

Women with diabetes were recruited from the Department of Obstetrics and Gynecology, Mie University Hospital. The subjects were examined at the Department of Ophthalmology during the gestational period.

### Control, non-pregnant diabetic women (Group IV)

Women with diabetes were recruited from the Department of Endocrinology, Mie University Hospital.

For the pregnant groups, blood samples were collected at the regular prenatal checkups which ranged from 5 to 37 weeks. For the other groups, blood samples were collected at no specific times. The serum glucose level was determined by the hexokinase glucose-6-phosphate dehydrogenase method. Venous blood was drawn into plastic tubes containing EDTA as an anticoagulant, and plasma was obtained by centrifugation at 400 x g for 10 min and were stored at -80° C. The thawed plasma was centrifuged at 400 x g for 10 min before the measurements. Body mass index (BMI) was also estimated from the height and body weight at the sample collection.

### ELISA assays

ELISA assays were used to quantify the levels of the bioactive molecules in the vitreous samples with the commercial human VEGF, sVEGF R-1, and soluble form of sVEGF R-2 ELISA kits (R&D Systems, Minneapolis, MN). Each assay was performed in duplicate in accordance with the manufacturer’s instructions.

### Measurements of cytokines and growth factors

The plasma concentrations of various cytokines, e.g., eotaxin, granulocyte macrophage colony stimulating factor (GM-CSF), interferon (IFN)-γ, interleukin (IL)-1B, IL-2, IL-4, IL-6, IL-8, IL-10, IL-17, monocyte chemotactic protein (MCP) -1 and tumor necrosis factor (TNF)-α were measured with the Luminex Multiplex Assay (Luminex Corporation, Austin, TX). Multianalyte profiling was performed with the Luminex-100 system and the XY Platform. Calibration microspheres for classification and reporter readings as well as sheath fluid were purchased from Luminex Corporation. Acquired fluorescence data were analyzed by the Master Plex ™ QT software (Ver. 1.2, Mirai Bio, Inc. San Bruno, CA). All analyses were performed according to the manufacturer’s protocols.

### Ophthalmic examinations

Fundus examinations of the patients were performed by trained retinal specialists. The examination of Group II was performed at the initial and late term stage of pregnancy. The examination of Group III was offered after their first antenatal clinic visit, and 28 weeks after the pregnancy and late term. The examinations of Group I and IV were performed as recommended. A progression of DR was defined as the advancement of one stage of DR in at least one eye. Patients with proliferative DR changes were excluded from the study.

### Statistical analyses

All experiments were repeated at least three times and values are presented as the means ± standard deviations. Data were analyzed by one-way non-repeated ANOVA followed by Bonferroni post-hoc tests for the comparison of the means. Spearman’s rank-order correlation coefficient was used to determine the significance of the correlations among the variables. The strength of the correlation (r-value) was classified as: 0.0 to 0.2 not correlated or very weak; 0.2 to 0.4 weak or low; 0.4 to 0.7 moderate; 0.7 to 0.9 strong or high; and 0.9 to 1.0 very strong. Statistical significance was set at *P* <0.05.

## Results

### Pregnancy alters level of human VEGF, sVEGFR-1, and sVEGFR-2

The demographic data of the study subjects in the four groups are shown in Table 1. No significant differences were observed in the age,gestational weeks, and the BMI among the groups. The blood sugar level was significantly lower in Group II than in Groups III and IV (*P* <0.05; non-repeated ANOVA). No progression of the DR was detected during the study period in any of the groups.

**Table 1:** Demographic data of study subjects.

	Group I	Group II	Group III	Group IV	P
DM	-	+	+	+	
Pregnant	+	+	+	-	
No. of patients	10	19	16	22	n.s
Age (yrs)	31.2 ± 3.3	35.8 ± 5.0	33.5 ± 5.8	35.5 ± 4.2	n.s
Gestational age (wks)	27.9 ± 5.7	24.6 ± 6.2	24.8 ± 9.4	(-)	n.s
Blood glucose	(-)	123.0 ± 40.2*	208.1 ± 96.0	163.5 ± 69.5	n.s
BMI (%)	23.1 ± 4.1	25.4 ± 6.2	24.5 ± 2.5	27.6 ± 5.2	n.s

Data are the means ± standard deviations. Non-repeated ANOVA was used to determine the significance of the correlation between the groups. Group I, normal pregnancy; Group II, gestational diabetes mellitus; Group III, preexisting diabetes; Group IV, diabetic women without pregnancy. BMI: body mass index. n.s, not significant.

*P < 0.05.

**Fig 1:**
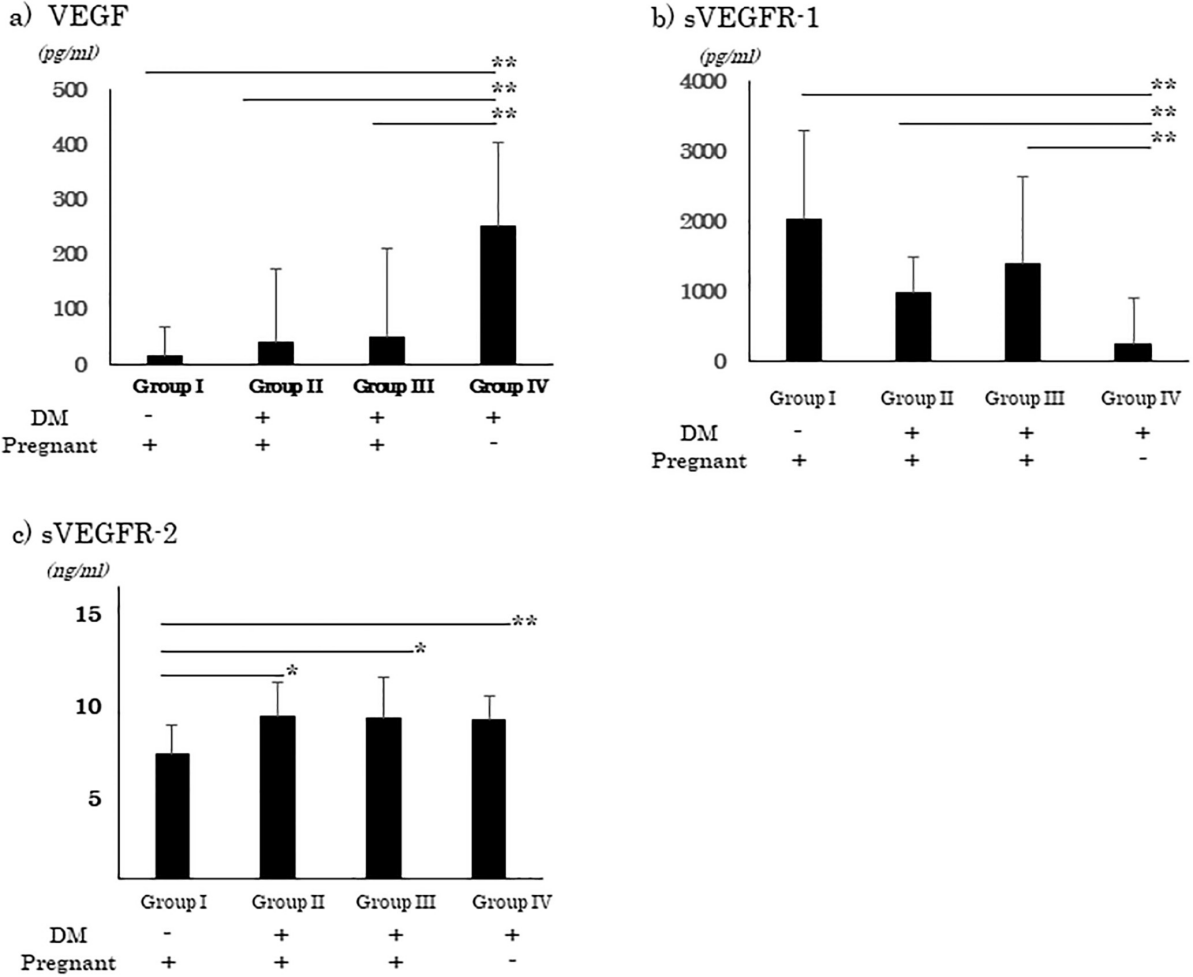
Plasma concentration of VEGF, sVEGF-R1, and sVEGF-R2 during pregnancy. The plasma concentrations of VEGF, sVEGF-R1, and sVEGF-R2 were estimated by ELISA for each group. The VEGF concentration is significantly lower in Group I, Group II, and Group III than in Group IV (a). The sVEGFR-1 is significantly higher in Group I than in Group II, Group III, and Group IV (b). The sVEGFR-2 is significantly higher in the Group II, Group III, and Group IV than in Group I (c). Group I, normal pregnancy; Group II, women with GDM; Group III, women with preexisting diabetes; and Group IV, diabetic non-pregnant women. VEGF, vascular endothelial growth factor; sVEGFR-1, soluble form of VEGF receptor-1; sVEGFR-2, soluble form of VEGF receptor-2. *: *P* <0.05, **: *P* <0.01, non-repeated ANOVA.

The VEGF concentration was significantly lower in Group I (16.3 ± 51.6 pg/μl), Group II (41.5 ± 131.8), and Group III (49.0 ± 160.9) than in Group IV (251.4 ± 152.7, *P* <0.01; Fig 1a). Thus, the concentration of VEGF was lower during pregnancy. On the other hand, the concentration of plasma sVEGFR-1, was significantly lower in Group IV (241.6 ± 652.2 pg/μl) than in the pregnant groups; Group I (2023.8 ± 1273.2 pg/μl), Group II (970.7 ± 514.4 pg/μl) and Group III (1392.9 ± 1237.4 pg/μl, *P* <0.01, Fig 1b). The concentration of plasma sVEGFR-2 was significantly lower in Group I (6.4 ± 1.5 ng/μl) than in the DM-associated groups; Group II (8.3 ± 2.1 ng/μl), Group III (8.3 ± 2.1 ng/μl), and Group IV (8.2 ± 1.3 ng/μl, *P* <0.05, Fig 1c).

### Inflammatory cytokines are altered during CMDP

**Fig 2:**
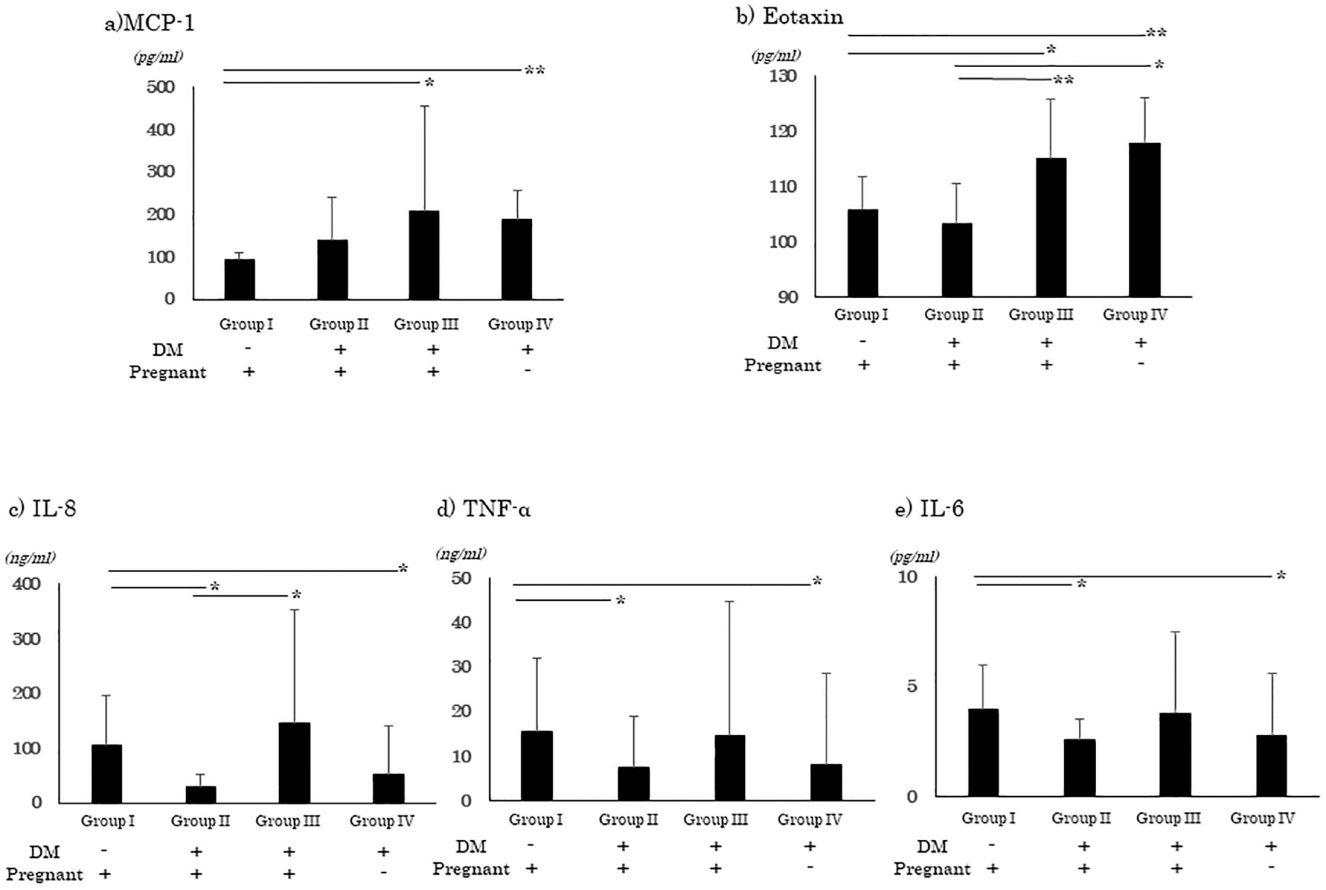
Alterations of inflammatory cytokines during pregnancy. Various inflammatory cytokines were evaluated using a multiplex system. MCP-1 is lower with Group I than in Group III and Group IV (a). Eotatin is lower in Group I than in Group III and Group IV (b). IL-8 is significant higher in Group I than in Group II and Group IV (c). TNF-α is significant higher in Group I than in Group II and Group IV (d). IL-6 is significant higher in Group I than in Group II and Group IV (e). Group I, normal pregnancy; Group II, women with GDM; Group III, women with preexisting diabetes; and Group IV, diabetic non-pregnant women. IL, interleuikin; MCP-1, monocyte chemotactic protein-1; TNF-α, tumor necrosis factor-α. *: *P* <0.05, **: *P* <0.01, non-repeated ANOVA.

Because VEGF and sVEGFR-1 have been shown to be associated with inflammation, we also determined the concentrations of the other plasma inflammatory cytokines for the four groups of women using a multiplex system. The results showed that IL-1B, IL-2, IL-4, IL-10, IL-17, IFN-γ, and GM-CSF were not significantly different among the groups. The level of MCP-1 was significantly lower in Group I (94.6 ± 13.7 pg/ml) than in Group III (206.7 ± 248.9 pg/ml, *P* <0.05) and Group IV (188.8 ± 67.0 pg/ml, *P* <0.01, Fig 2a). A similar pattern was found for eotaxin. Eotaxin was significantly lower in Group I (105.8 ± 6.0 pg/ml) than in Group III (115.0 ± 10.9 pg/ml, *P* <0.05) and in Group IV (117.9 ± 8.1 pg/ml, *P* <0.01, Fig 2b). Thus, MCP-1 and eotaxin tended to be lower in Group I than in Groups III and IV. There appeared to be a tendency for the concentration of MCP-1 and eotaxin to be lower during pregnancy which is similar to the pattern of VEGF.

IL-8 was significant higher in Group I (105.7 ± 91.2 pg/ml) than in Group II (30.7 ± 21.6 pg/ml) and Group IV (52.9 ± 88.5 pg/ml, Fig 2c). TNF-α was also significant higher in Group I (15.5 ± 16.6 pg/ml) than Group II (7.6 ± 11.5 pg/ml) and Group IV (8.0 ± 20.5 pg/ml, *P* <0.05, Fig 2d). IL-6 was significant higher in Group I (4.0 ± 2.1 pg/ml) than in Group II (2.6 ± 0.9 pg/ml) and Group IV (2.77 ± 2.82 pg/ml, *P* <0.05, Fig 2e). Thus, IL-8, TNF-α, and IL-6 tended to be higher in Group I than in the other groups which is different from the pattern of VEGF but similar to the pattern of sVEGFR-1.

### Correlation between the VEGF-related molecules and the inflammatory cytokines (Table 2)

We also determined the correlations between the concentrations of the VEGF-related molecules, VEGF, sVEGFR-1, and sVEGFR-2, and the concentrations of the inflammatory cytokines for each group. For group I, a strong correlation was found between VEGF and sVEGF-R1 (Spearman’s rank-order correlation coefficient, r = 0.79, *P* <0.01), a moderate correlation was observed between sVEGF-R1 and TNFα (r = 0.64, *P* <0.05), sVEGF-R2 and MCP-1 (r = 0.57, *P* <0.05).

**Table 2:** The correlation between VEGF-related molecules and inflammatory cytokines.

(r value)		Eotaxin	MCP-1	IL-8	TNF-α	IL-6
	VEGF	n.s	n.s	n.s	n.s	n.s
Group I	sVEGFR-1	n.s	n.s	n.s	0.64*	n.s
	sVEGFR-2	n.s	0.57*	n.s	n.s	n.s
	VEGF	n.s	n.s	n.s	n.s	n.s
Group II	sVEGFR-1	n.s	n.s	0.45*	n.s	0.43*
	sVEGFR-2	n.s	n.s	n.s	n.s	n.s
	VEGF	n.s	n.s	n.s	n.s	n.s
Group III	sVEGFR-1	n.s	n.s	n.s	n.s	0.47*
	sVEGFR-2	n.s	0.53*	n.s	n.s	0.38*
	VEGF	n.s	n.s	n.s	n.s	n.s
Group IV	sVEGFR-1	n.s	n.s	n.s	n.s	n.s
	sVEGFR-2	n.s	n.s	n.s	n.s	n.s

Spearman’s rank-order correlation coefficient was used to examine the relationship among the variables. IL, interleukin; MCP-1, monocyte chemotactic protein-1; sVEGFR-1, soluble form of VEGF receptor-1; sVEGFR-2, soluble form of VEGF receptor-2; TNF-α, tumor necrosis factor-α; VEGF, vascular endothelial growth factor. Group I, normal pregnancy; Group II, gestational diabetes mellitus; Group III, preexisting diabetes; Group IV, diabetic women without pregnancy

*P < 0.05.

For group II, a weak correlation was found between sVEGFR-1 and IL6 (r = 0.43, *P* <0.05), and between sVEGFR-1 and IL-8 (r = 0.45, *P* <0.05). For group III, a moderate correlation was found between sVEGFR-1 and MCP-1 (r = 0.53, *P* <0.01), between sVEGFR-1 and IL-6 (r = 0.47, *P* <0.05), and between sVEGFR-2 and IL-6 (r = 0.38, *P* <0.05). For group IV, a strong correlation was found only between VEGF and sVEGFR-1 (r = 0.84, *P* <0.01).

## Discussion

The results showed that the pattern of expression of the VEGF-related molecules and the inflammatory cytokines were different for CMDP patients compared with regular DM or pregnant patients. To maintain a stable pregnant condition, various endocrine or immune systems must function normally. Our results showed the complicated manner in the expression pattern of various molecules in CMDP. We observed that the level of VEGF was not elevated in the CMDP group although it was significantly elevated in the DM group, and the concentrations of sVEGFR-1 and sVEGFR-2 were also different among the groups. We also found that the ratio of sVEGR1/2: VEGF was significant higher for the DM group than the CMDP groups (S1 Fig). These results indicate that the free VEGF levels were significantly lower in patients with CMDP.

Angiogenesis and inflammation are two highly linked processes, and they induce endothelial cell disfunction. In the last two decade, many factors with dual functions in both of these major pathways have been identified including the cytokines we studied here. Immune cells play a critical role in regulating wound healing and in controlling neovascularization. The immune cells that have been implicated in vascular formation include macrophages, dendritic cells, granulocytes, and lymphocytes. These cells play different roles in the early stages of angiogenesis. [26]. The results showed that the level of VEGF-related molecules and the cytokines were different with CMDP and relate to endothelial disfunction. Most of them are related to angiogenesis and endothelial cell. MCP-1 is a CC chemokine which can directly mediate angiogenesis. Previous study demonstrated that MCP-1induced migration of cultured endothelial cell migration in a dose-responsive manner p [27]. Eotaxin, another CC chemokine, was also reported to mediate angiogenic responses through the CCR3 receptor expressed by human microvascular endothelial cells [28]. IL-6 is an inflammatory cytokine secreted by a variety of cell types including fibroblasts, monocytes, and endothelial cells. It is also associated with other inflammatory factors, such as TNF-α, IL-10, IL-18, MCP-1, intercellular adhesion molecule-1, and vascular cell adhesion molecule-1 [29, 30]. TNF-α is an inflammatory mediator affects the early stages of carcinogenesis, including angiogenesis and invasion [31]. Finally, IL-8 also has a role in angiogenesis, with production from endothelial cells, leucocytes and fibroblasts resulting in capillary tube formation, endothelial cell proliferation [32, 33]. We found a significant difference among these molecules for CMDP and the correlation between VEGF-related molecules. However, our multiplex results showed no significance between the groups with other molecules, e.g. GM-CSF, IFN-γ, IL-1B, IL-2, IL-4, IL-10 and IL-17. This implies the complexity of the balance between inflammation and angiogenesis with endothelial cell damage with CMDP. Because the CMDPs are in the mixed status of pregnancy and diabetic changes, their pathological backgrounds are complicated and many circulating factors including cytokines may alter them. Our results indicate that the combination of these molecules strengthen the inflammatory changes, and it implies that this is the origin of complications during CMDP.

Pregnancy is known to be one of the risk factors for the progression of DR. The rate of DR progression occurs at approximately twice the rate during pregnancy than non-pregnancy [34]. Any grade of DR occurs in 12.7% and proliferative DR occurs in 4.2% of insulin-dependent pDM patients [35]. Micro vascular examinations during pregnancy have shown that the odds ratio for DR progression in pregnant women was significantly higher in the conventionally-treated group than in the intensely-treated group [36]. VEGF is also known to be a key molecule expressed in eyes with retinal hypoxia or ischemia and can cause micro-circulatory changes including neovascularization resulting in DR progression [7, 8]. The VEGF concentration in the aqueous fluid of DM patients is higher than that in controls [37], and it is significantly correlated with the severity of DR. The processes of retinal ischemia and vascular remodeling are reported to be associated with inflammation [38, 39]. The interactions between the inflammatory cells and retinal cells have important relationships with intraocular neovascularizations. In vitreoretinal diseases, the vitreous levels of MCP-1, IL-6, and IL-8 are correlated which suggest a relationship between the inflammatory and angiogenic processes similar to our results [40–42].

We evaluated many kinds of factors in the human plasma and found that the levels of some of the cytokines, viz., MCP-1, eotaxin, IL8, TNFα, and IL6, were changed depending on the status of the pregnancy. Because there was no significant correlation between these molecules and VEGF for all groups, these changes are independent of that of VEGF. We can conclude that not only VEGF but other factors including these inflammation-related molecules may contribute to the diabetic changes with CMDP. In addition, it has been reported that sVEGFR-1 is involved in monocyte- or macrophage-related pathways, and it promotes inflammation more than sVEGFR-2 by recruiting or activating inflammatory cells [43, 44]. Our results showed that the action of sVEGFR-1 and sVEGFR-2 were different between the groups. To consider together with the action of the inflammatory molecules, there exists the importance of the inflammatory aspects of CMDP.

Among the patients with CMDP, GDM is also a potential risk factor. Cohort data from 893 eligible women from 30 institutions in Japan, their background GDM was found to impact the fetal growth [45]. But from 9888 cohort, patients with GDM had a significantly higher incidence of ophthalmic morbidity compared with controls, and the authors concluded that GDM is an independent risk factor for long-term maternal ophthalmic morbidity [46]. In addition, we observed significant correlation between various inflammatory cytokines (MCP-1, IL6 and IL8) and sVEGFR-1 for the pDM/GDM group. These differences may contribute to the complications in GDM patients during pregnancy. And because the dynamics of these molecules are not necessarily the same between GDM and pDM, these results also confirm the different pathophysiology between them.

There are limitations in this study including the small number of patients in each group. First, there exists the possibility that the use of insulin or glycemic control may have affected the results because of the association between inflammation and increased risk of type 2 diabetes [47]. Insulin therapy itself exacerbates the diabetic blood-retinal barrier breakdown in the presence of VEGF [48]. There were 7 patients in the GDM group, 16 in the pDM group, and 18 patients in the diabetes group who were undergoing insulin therapy during the time of this study. Although, we also compared VEGF, sVEGFR-1 and sVEGFR-2 among these group the received insulin therapy, the results were similar to that of all groups whether they received insulin or not (S2 Fig). We conclude that we could not find any insulin influence on our results. Second, there is a possibility that the time of sample collection, the gestational age, may have affected the results including that of VEGF. The VEGF level may depend on the time of sample collection because as blood vessel formation progresses, hypoxia progressively decreases which then leads to a reduction in the production of VEGF at the end of gestation. And finally, we studied only DM patients whose disease was well controlled without major complications, and none of the patients had signs of progression to severe DR. Clinically, the Diabetic Retinopathy Preferred Practice Pattern from the American Academy of Ophthalmology stated that,

*“If any DR exist at the first antenatal clinic appointment*, *additional retinal assessment is recommended at 16 to 20 weeks*, *but we do not need assessment until 28 weeks if no DR at the initial examination”* [49].

This indicates that the progression of DR in patients with CMDP is dependent on the baseline status of DR. There existed a selection bias in our study, and there is a possibility that VEGF may increase in patients with poor blood sugar control or severe DR. This is because diabetes education and control programs are very well instituted, and fewer patients suffer from severe DR during their pregnancy [50].

In conclusion, our findings indicate that the VEGF-related molecules and the inflammation-related molecules are involved in the complications in CMDP. However, further investigations into the relationship between VEGF and inflammation-related molecules and complications, including DR progression in CMDP need to be performed.

## Supporting information

S1 Fig Ratio of plasma VEGF and sVEGF-R1, sVEGF-R2 during pregnancy. The ratio of plasma VEGF and sVEGF-R1 (VEGF/sVEGF-R1) and VEGF and sVEGF-R2 (VEGF/sVEGF-R2) were estimated for each group. The VEGF/sVEGF-R1 is significantly lower in Group I, Group II, and Group III than in Group IV (a). The VEGF/sVEGF-R2 is significantly lower in Group I, Group II, and Group III than in Group IV (b). Group I, normal pregnancy; Group II, women with GDM; Group III, women with preexisting diabetes; and Group IV, diabetic non-pregnant women. VEGF, vascular endothelial growth factor; sVEGFR-1, soluble form of VEGF receptor-1; sVEGFR-2, soluble form of VEGF receptor-2. **: P <0.01, non-repeated ANOVA.
(TIF)

S2 Fig Plasma concentration of VEGF, sVEGF-R1 and sVEGF-R2 during pregnancy treated with insulin. The plasma concentrations of VEGF, sVEGF-R1, and sVEGF-R2 of the patients treated with insulin were estimated by ELISA for each group. The VEGF concentration of the patients treated with insulin is significantly lower in Group I, Group II, and Group III than in Group IV (a). The sVEGFR-1 of the patients treated with insulin is significantly higher in the Group I than in Group II, Group III, and Group IV (b). The sVEGFR-2 of the patients treated with insulin is significantly higher in the Group II, Group III, and Group IV than in Group I (c). Group I, normal pregnancy; Group II, women with GDM; Group III, women with preexisting diabetes; and Group IV, diabetic non-pregnant women. VEGF, vascular endothelial growth factor; sVEGFR-1, soluble form of VEGF receptor-1; sVEGFR-2, soluble form of VEGF receptor-2. *: P <0.05, **: P <0.01, non-repeated ANOVA.
(TIF)
