# Supplementary figures and images for: Changes in VEGF-related factors are associated with presence of inflammatory factors in carbohydrate metabolism disorders during pregnancy
Source: PLoS One. 2019 Aug 15;14(8):e0220650. doi: 10.1371/journal.pone.0220650 (PMC6695137; doi:10.1371/journal.pone.0220650)

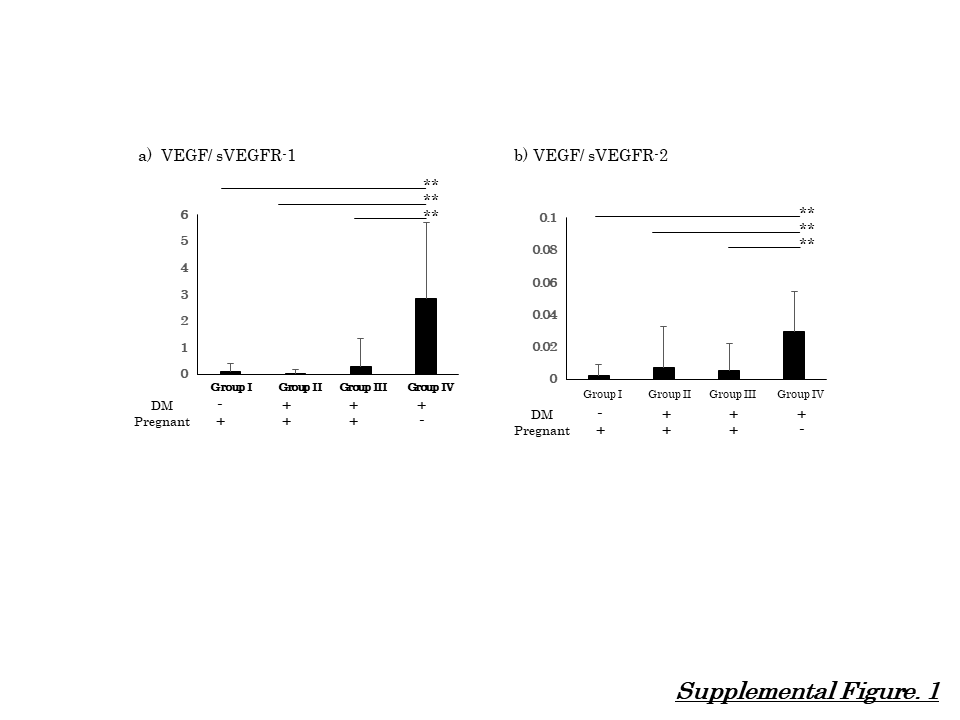

Supplement: S1 Fig — The ratio of plasma VEGF and sVEGF-R1 (VEGF/sVEGF-R1) and VEGF and sVEGF-R2 (VEGF/sVEGF-R2) were estimated for each group. The VEGF/sVEGF-R1 is significantly lower in Group I, Group II, and Group III than in Group IV (a). The VEGF/sVEGF-R2 is significantly lower in Group I, Group II, and Group III than in Group IV (b). Group I, normal pregnancy; Group II, women with GDM; Group III, women with preexisting diabetes; and Group IV, diabetic non-pregnant women. VEGF, vascular endothelial growth factor; sVEGFR-1, soluble form of VEGF receptor-1; sVEGFR-2, soluble form of VEGF receptor-2. **: P <0.01, non-repeated ANOVA. (TIF) [file pone.0220650.s001.TIF]

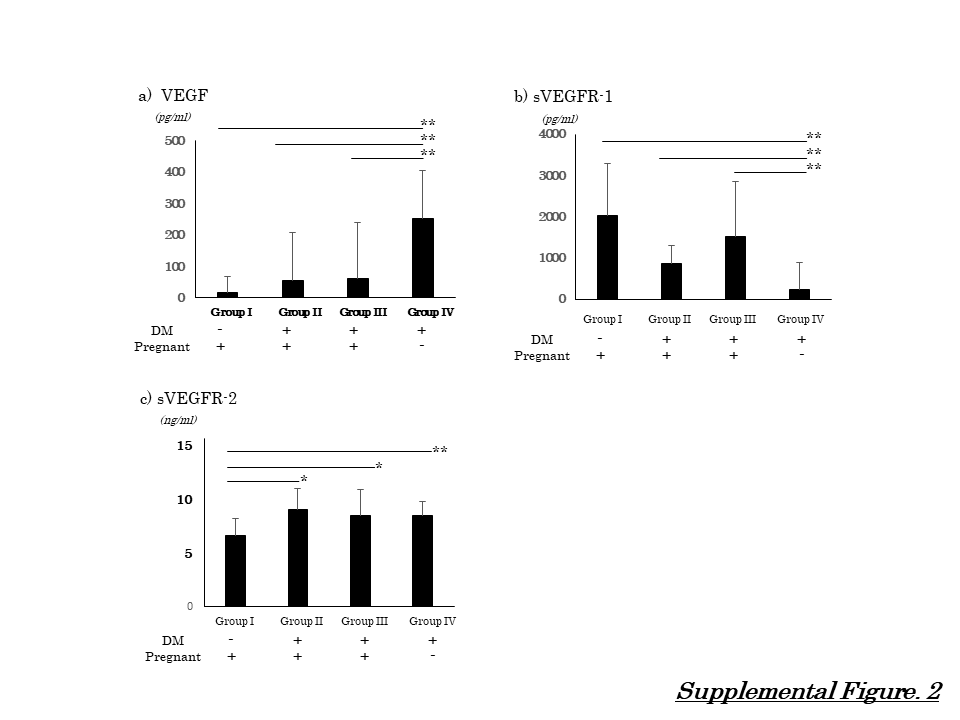

Supplement: S2 Fig — The plasma concentrations of VEGF, sVEGF-R1, and sVEGF-R2 of the patients treated with insulin were estimated by ELISA for each group. The VEGF concentration of the patients treated with insulin is significantly lower in Group I, Group II, and Group III than in Group IV (a). The sVEGFR-1 of the patients treated with insulin is significantly higher in the Group I than in Group II, Group III, and Group IV (b). The sVEGFR-2 of the patients treated with insulin is significantly higher in the Group II, Group III, and Group IV than in Group I (c). Group I, normal pregnancy; Group II, women with GDM; Group III, women with preexisting diabetes; and Group IV, diabetic non-pregnant women. VEGF, vascular endothelial growth factor; sVEGFR-1, soluble form of VEGF receptor-1; sVEGFR-2, soluble form of VEGF receptor-2. *: P <0.05, **: P <0.01, non-repeated ANOVA. (TIF) [file pone.0220650.s002.TIF]
